# Charity's Financial Outlook

**Published:** 1889-12-07

**Authors:** 


### Fallow Crops.

### CHARITY'S FINANCIAL OUTLOOK.

Upon all sides we hear of revival of trade. Men of business no longer deny a present improvement or shake their heads gloomily over the prospect of the future. "I believe we are fairly started on a period of great prosperity," said Mr. Chamberlain lately, and he added that "all classes alike will share in the harvest." We wonder if "in all classes" he would include the great charitable institutions of the country, many of which have had a hard struggle to survive the long period of depression, fraught for them with both increased work and diminished resources. The chances are that they were not in the speaker's mind, yet is it too much to hope that better trade will mean a more generous support for our hospitals? In these columns hospitals are naturally awarded premier place, and we claim that the cure of its sick poor is alike the first philanthropic duty of a civilised community, and an initial requirement of sound political economy. To be prosperous, a people must be healthy; and if it be conceded that medical science is instrumental in promoting health, whence does medical science draw its inspirations and garner its knowledge if not in our hospitals?

It may suit the busy man, who boasts that he never had a day's illness, to shut his eyes to some obvious facts, outside his control, which have conduced to his immunity, or it may appear reasonable to a certain class of philosophers to denounce our hospitals as pauperisers, but Dr. Johnson's words of 130 years ago are as true now as when they were uttered, and for arguments—if any were needed—in support of our hospitals, *The Idler* of May 6, 1758, would be powerful and appropriate still.

The support of our charitable institutions remains the adopted privilege of the few, and not until the public of all classes, those who directly and those who indirectly benefit by their work, recognise an obligation and act up to their sense of duty will the hospital question be solved by voluntary effort.

Similar observations might be applied to some other charitable endeavours where work is crippled and rendered imperfect for want of a more general and a more spontaneous support. But while the co-operation of all classes in, what we may term, "day-by-day" charity is desirable, there is a colossal philanthropy essentially personal in its origin, and only possible to the wealthy, of which we need a few more examples, such as have been recently supplied by Sir Edward Guinness and Sir Sydney Waterlow.

Whatever the cause, there can be no doubt in the minds of those who examine these matters closely that as a body the very rich fail to act up to their responsibilities, and exhibit an indifference and selfishness calculated in these democratic days to bring speedy retribution were it not that their shortcomings are palliated, and in a way covered by the interposition of a less wealthy and more self-sacrificing class. Even when leaving the world these millionaires grudge mankind a share of the wealth they can no longer keep, and with a perversity little short of criminal add all to family hoards already overflowing.

It is a healthy sign that a paper like the *Standard* has the courage to utter a few plain truths in the ears of the classes whose interests especially it is supposed to safeguard. Time was when a Conservative paper would have hesitated before reminding the great landlords of London that in these matters they have been weighed and are found wanting. "The old nobility"—we ourselves should not limit the reproach to them—"apparently do not find that rank is exacting in its demands on the treasure chest. Yet it is they, of all others, who should feel the strongest compulsion of honour." Not long ago, criticism of this kind would have been deemed better suited to the columns of some Radical or Socialistic print, where it would have been read only by those whom it did not concern.

It is this holding aloof of those who are most capable of assisting—upon whom rests most heavily the obligation to assist—which is the saddest feature of our social system. It makes true constitutionalists despair, and is without doubt sowing the seeds of serious trouble and possible revolution. Contrasted with a depth of misery which has had few parallels in any city and in any period of the world's history, there is an ostentatious display of luxurious self-indulgence which long ago must have amassed a dangerous spirit of resentment in any but a spiritless multitude.

But we know that the direct relief of a poverty-stricken population born and bred among unwholesome surroundings, and not only poor but too often vicious, is an undertaking fraught with stupendous difficulties, and an individual, however potent and philanthropic, may well pause before essaying it. The indirect methods followed by the benefactors we have named are wise, and in a measure simple. To provide better homes and means of healthful recreation are steps in the direction of important results, but we need other efforts converging upon the same goal.

The accepted public institutions of the metropolis offer a safe field for benevolent enterprise, but how do they fare? The year records not one single act of individual munificence; something to impress the imagination with a sense of its sufficiency and power. The hospitals looked to for the performance of indispensable duties either totter under a load of debt, or seek to curtail their work as a sorry method of paying their way. The *Standard* deplores "the absence of a sense of the duties which property entails." Upon the particular class to whom this observation applies rests a heavy and peculiar obligation. Their property has grown to its present enormous proportions by no act of theirs. Theirs is not even the merit of prevision. Inherited at the outset, their possessions have been increased by the industry and exertions of anyone rather than themselves, and the very causes which have quadrupled their wealth have called into being whole communities of sick and hungry people. Yet their united contribution to the hospitals of the metropolis is infinitesimal as compared with the expenditure of the institutions, and impalpable when considered as a portion of their own enormous incomes. Well may we share the regret of our contemporary that the grand old maxim "Noblesse oblige" has ceased to inspire.

Agricola.

